# OnmiMHC: a machine learning solution for UCEC tumor vaccine development through enhanced peptide-MHC binding prediction

**DOI:** 10.3389/fimmu.2025.1550252

**Published:** 2025-02-28

**Authors:** Fangfang Jian, Haihua Cai, Qushuo Chen, Xiaoyong Pan, Weiwei Feng, Ye Yuan

**Affiliations:** ^1^ Department of Obstetrics and Gynecology, Ruijin Hospital, Shanghai Jiao Tong University School of Medicine, Shanghai, China; ^2^ DigitalGene, Ltd, Shanghai, China; ^3^ Institute of Image Processing and Pattern Recognition, Shanghai Jiao Tong University, and Key Laboratory of System Control and Information Processing, Ministry of Education of China, Shanghai, China; ^4^ Key Laboratory of Biopharmaceutical Preparation and Delivery, Chinese Academy of Sciences, Beijing, China; ^5^ State Key Laboratory of Biochemical Engineering, Institute of Process Engineering, Chinese Academy of Sciences, Beijing, China

**Keywords:** peptide-MHC binding, MHC I and II, deep learning, uterine corpus endometrial carcinoma, neoantigen

## Abstract

The key roles of Major Histocompatibility Complex (MHC) Class I and II molecules in the immune system are well established. This study aims to develop a novel machine learning framework for predicting antigen peptide presentation by MHC Class I and II molecules. By integrating large-scale mass spectrometry data and other relevant data types, we present a prediction model OnmiMHC based on deep learning. We rigorously assessed its performance using an independent test set, OnmiMHC achieves a PR-AUC score of 0.854 and a TOP20%-PPV of 0.934 in the MHC-I task, which outperforms existing methods. Likewise, in the domain of MHC-II prediction, our model OnmiMHC exhibits a PR-AUC score of 0.606 and a TOP20%-PPV of 0.690, outperforming other baseline methods. These results demonstrate the superiority of our model OnmiMHC in accurately predicting peptide-MHC binding affinities across both MHC-I and MHC-II molecules. With its superior accuracy and predictive capability, our model not only excels in general predictive tasks but also achieves significant results in the prediction of neoantigens for specific cancer types. Particularly for Uterine Corpus Endometrial Carcinoma (UCEC), our model has successfully predicted neoantigens with a high binding probability to common human alleles. This discovery is of great significance for the development of personalized tumor vaccines targeting UCEC.

## Introduction

The MHC is a crucial component of the immune system, with MHC-I and MHC-II molecules each playing a key role in antigen presentation and specific immune responses. MHC-I molecules are mainly present on the surface of all nucleated cells and are responsible for presenting antigen peptides produced within the cell to CD8+ T cells, while MHC-II molecules are widely present on the surface of immune cells in humans and other vertebrates, such as antigen-presenting cells, which are responsible for processing foreign antigens into peptides and presenting them to CD4+ T cells (1–5).

For investigating the binding of MHC-I and MHC-II molecules to peptides, traditional biochemical experimental methods are accurate but time-consuming and labor-intensive, and there are certain technical limitations. With the development of machine learning technology, prediction methods based on machine learning have gradually become mainstream (6, 7). These methods use large volume of known data sets to train models to predict the possibility of new peptides binding to MHC molecules (8–12).

At present, various computational methods have been developed to predict the binding of peptides to MHC molecules, which is a crucial step in understanding the immune response. These methods can be broadly categorized into one group relying on binding affinity (BA) data and the other group utilizing mass spectrometry (MS) experimental data. For instance, NetMHCpan and NetMHCIIpan are well-established tools that have been recently updated to versions 4.1 and 4.0, respectively. These tools employ machine learning strategies to integrate different types of training data, including BA and MS-derived eluted ligand (EL) data, resulting in state-of-the-art performance (13, 14). Other notable approaches include MHCflurry, an open-source tool that predicts Class I MHC binding affinity and has been recognized for its accuracy and speed. Additionally, there are methods like MHCnuggets, which apply deep learning to predict neoantigens presented by MHC Class I and II molecules (15, 16).

Despite the advancements, these methods still have room for improvement, particularly in terms of their generalizability in handling the complexity of the antigen presentation. Some models may struggle with the inherent biases present in the training data, such as the overrepresentation of certain peptide features or the underrepresentation of others due to experimental limitations.

To address these challenges and further enhance the prediction accuracy, we proposed a novel deep learning approach, OnmiMHC. Our method integrates BA data with MS experimental data, leveraging the strengths of both to provide a more comprehensive and robust prediction model. By employing advanced neural network architectures and training strategies, OnmiMHC aims to improve upon the current state-of-the-art by offering higher accuracy, broader applicability, and better generalization across diverse MHC-peptide interactions. In this study, we first used BA data to construct a deep learning pre-training model, which enabled the model to initially imitate the interaction rules between MHC molecules and peptide segments through the pre-training process. Then, we used the pre-trained model to annotate and filter the data detected by MS experiments, resulting in a high-quality dataset, which greatly expanded their training set. Through independent validation and comparative experiments with multiple other baseline methods, we demonstrated significant improvements in prediction accuracy and performance of OnmiMHC.

## Results

### BA-based multimodal data preprocessing and integration

**Figure 1:**
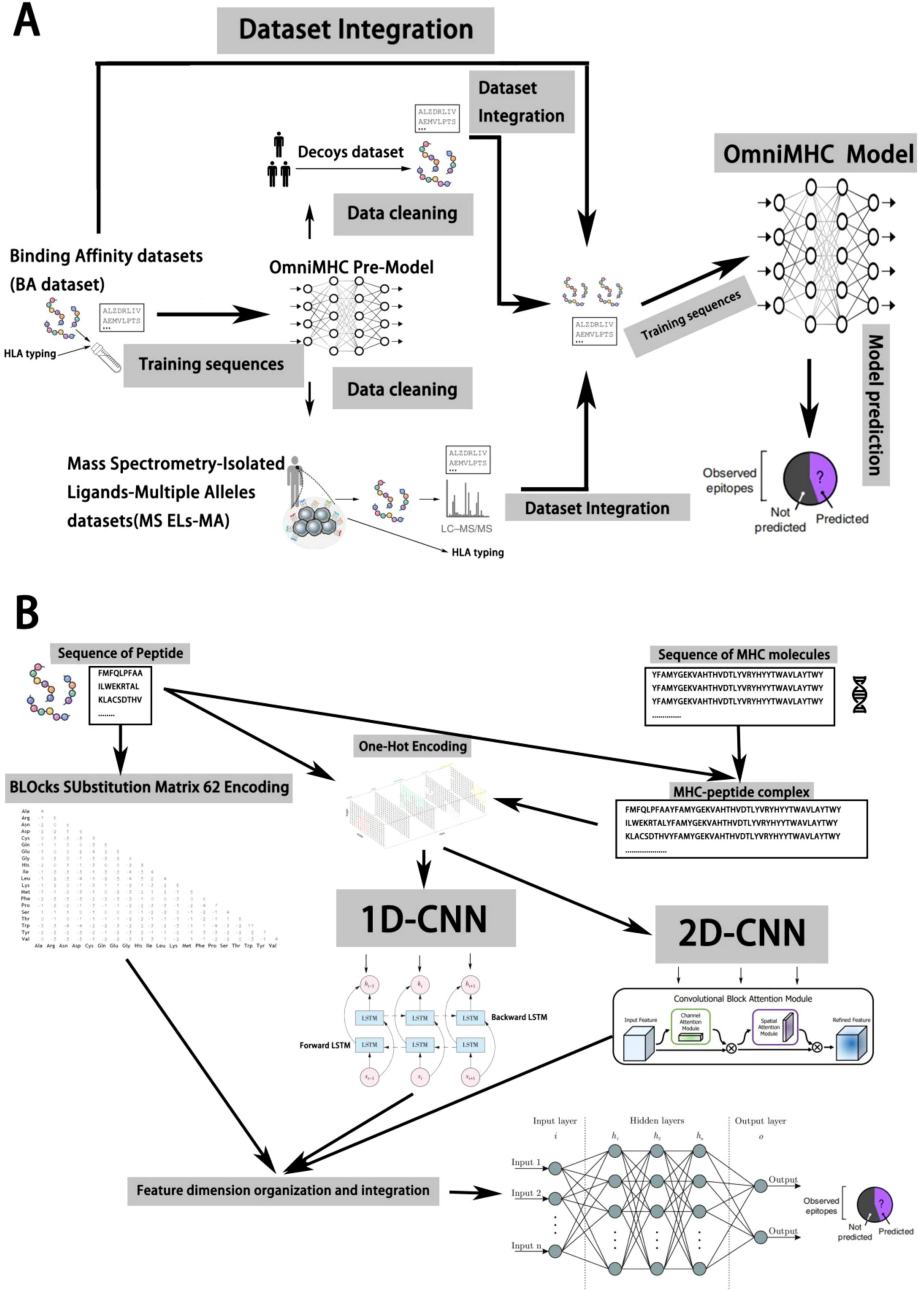
Model design and data preprocessing based on iterative methods. **(A)** We trained a regression model using Binding Affinity (BA) data from competitive binding experiments, then used this model to screen and refine Mass Spectrometry-Isolated Ligands datasets by eliminating outliers and improving the representation of non-binding samples, ultimately enhancing the reliability and accuracy of our model. **(B)** The OmniMHC model integrates 2D and 1D convolutional kernels, BiLSTM, CBAM, and Blosum62 encoding with concatenation to accurately predict MHC-peptide binding. This comprehensive design takes into account multiple features, thereby enhancing predictive performance.

Initially, we trained a regression model using the Binding Affinity (BA) dataset (14), which encompasses affinity data obtained from competitive binding experiments between peptides and specific allele proteins, scored by IC50 values. Subsequently, leveraging the trained regression model, we screened the Mass Spectrometry-Isolated Ligands-Single Allele (MS ELs-SA) and Mass Spectrometry-Isolated Ligands-Multiple Alleles (MS ELs-MA) datasets to eliminate potential outliers. These datasets were derived from experiments involving the dissociation process of ligands with MHC molecules. These experiments employ acidic solutions or alternative methods to dissociate antigenic peptide segments from MHC molecules, followed by identification and analysis via mass spectrometry or other techniques to obtain peptide sequences. Thus, these datasets solely cover positive samples capable of binding. While most experiments randomly select peptide sequences from the human body as negative samples, such practices lack rigor. Therefore, we utilized a pre-trained model to filter these negative samples, better representing non-binding data. For the MS ELs-MA dataset, we utilized a pre-trained model to predict each sample, labeling the allele protein with the highest score, thereby transforming multi-allele protein binding data into a single allele protein dataset. Through this process, we effectively integrated data from multiple experimental types, subsequently training models to enhance reliability and accuracy (refer to **Figure 1A**).

### Model design of OnmiMHC

Predicting the binding between MHC molecules and peptide segments is crucial for understanding immune responses. However, due to the complex interactions and high diversity between peptide segments and MHC molecules, it is technically challenging (1). In this study, we present a model named OnmiMHC to predict whether alleles and peptides can bind based on representations learned from the sequences of peptides and alleles.

The OmniMHC model adopts a strategy that integrates multimodal feature fusion, combining two-dimensional convolutional kernels with one-dimensional ones. After convolution, the features enter a BiLSTM for sequence information extraction, and Convolutional Block Attention Module2(CBAM) is introduced to attend to the features. Finally, various features and Blocks Substitution Matrix 62(Blosum62) (17) encoding are merged to capture the relationship between MHC molecules and peptide sequences. This model comprises the following components: (i) a two-dimensional convolutional neural network for extracting high-level abstract features from sequences of MHC molecules and peptides, (ii) a Convolutional Neural Network - Bidirectional Long Short-Term Memory(CNN-BiLSTM) (18) neural network for extracting binding sequences of MHC molecules and peptides, (iii) a CBAM module for attention on features, and (iv) lastly, through MLP, various features undergo dimensionality reduction to predict the binding between peptides and MHC molecules.

This design enables OmniMHC to comprehensively consider features from multiple perspectives and leverage the capabilities of neural network models to learn the binding probability between MHC molecules and peptide segments (**Figure 1B**). Through feature fusion, the model demonstrates superior performance.

### Model performance and comparison with other methods

To mitigate overfitting during the model training process, we adopted a 5-fold cross-validation method. This approach divides the data into five parts, and in each training iteration, four parts are used for training while the remaining one part is used for testing. This yields five models, and their predictions are averaged to obtain the final results. To ensure the rigorousness of the comparison experiments, we selected only models that provided complete training code for our experiments to ensure the consistent training and test set splits. Additionally, we performed online comparisons with multiple models on the IEBD platform.

For the MHC-I task, we utilized the public dataset of NetMHCpan-4.1, divided into five BA and five EL datasets. We performed five rounds of multimodal data preprocessing and integration based on BA., which includes 95 cell lines expressing individual HLA alleles created via stable transfection techniques and over 186,464 peptides binding to these HLA molecules (19).

For the MHC-II task, we employed the public dataset of NetMHCIIpan-4.1, also divided into five BA and five EL datasets. Similarly, we conducted five rounds of multimodal data preprocessing and integration based on BA, containing 81,422 unique HLA-DR401 peptides and 7,692 unique HLA-DR402 peptides identified through high-throughput screening methods based on yeast display technology (1, 20).

Finally, we conducted comparative experiments on the MHC-I and MHC-II tasks using the automated server benchmark datasets from the IEDB analysis resource (21, 22). These automated server benchmarks provide performance rankings for MHC-I and MHC-II servers and are regularly reassessed to stay updated. Each week, the latest version of IEDB automatically checks for sufficiently large datasets to add to these benchmarks, including BA datasets.

We used data from November 2022 to April 2024, which is relatively new and does not overlap with the training set. For the BA dataset in the test set, we classified samples with IC50 less than 500 nM as positive samples (high affinity) and the rest as negative samples, forming a classification test set.

OmniMHC achieves an area under precision-recall curve(PR-AUC) score of 0.854 and a TOP20%-PPV of 0.934 in the MHC-I task. These scores notably surpass those established models such as NetMHCpan4.1EL (14) (PR-AUC=0.729, TOP20%-PPV=0.670), mhcfurry-1.2.0 (15) (PR-AUC=0.600, TOP20%-PPV=0.593), and PickPocket (23) (PR-AUC=0.625, TOP20%-PPV=0.566)(**Supplementary File 1**).

**Figure 2:**
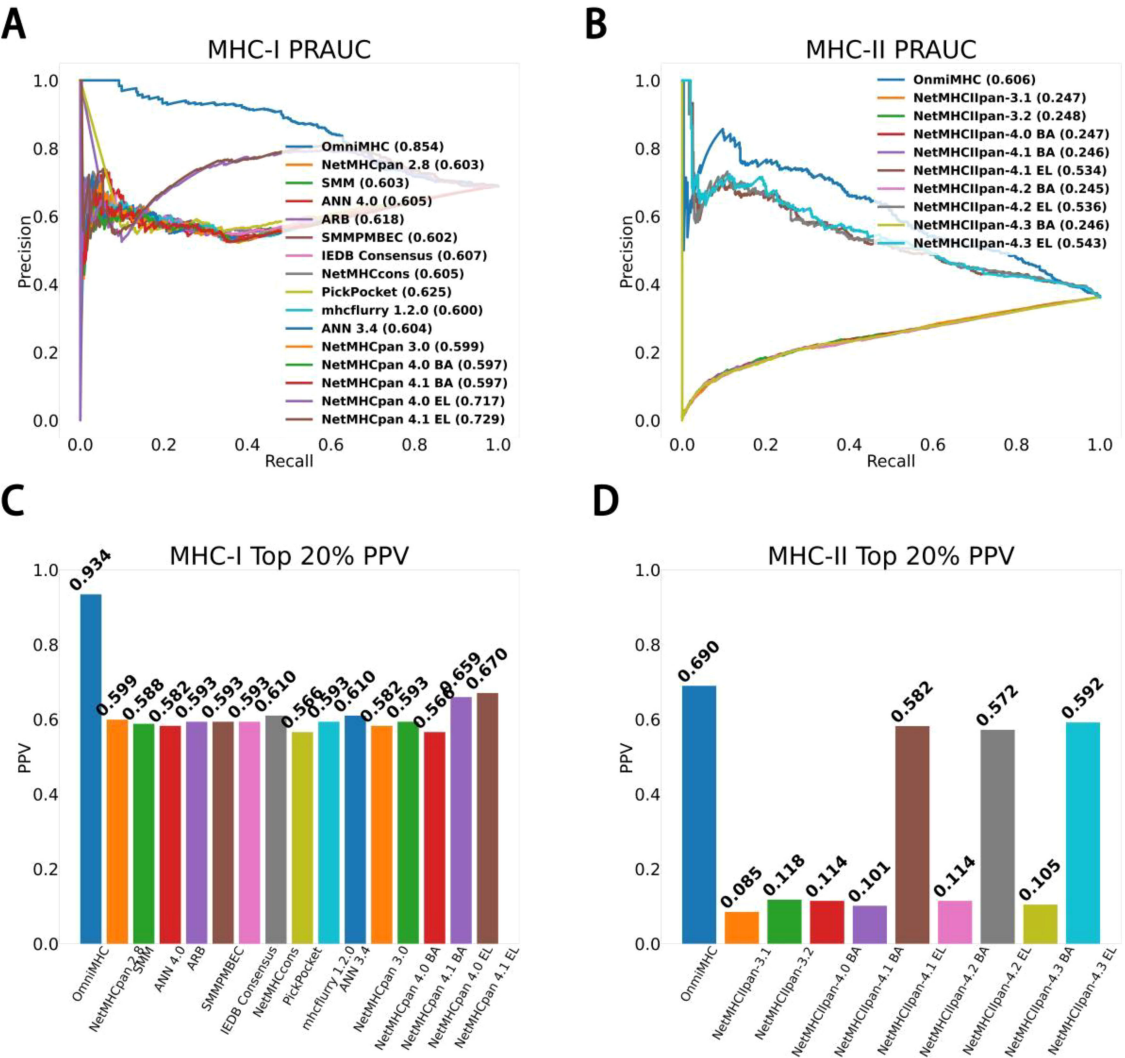
Model performance comparison. **(A)** MHC-I PR-AUC: The PR-AUC comparison for MHC-I tasks between OmniMHC and other models. **(B)** MHC-II PR-AUC: The PR-AUC comparison for MHC-II tasks between OmniMHC and other models. **(C)** MHC-I TOP20%-PPV: The TOP20%-PPV comparison for MHC-I tasks between OmniMHC and other models. **(D)** MHC-II TOP20%-PPV: The TOP20%-PPV comparison for MHC-II tasks between OmniMHC and other models.

Similarly, in the domain of MHC-II prediction, our model OmniMHC exhibited a PR-AUC score of 0.606 and a TOP20%-PPV of 0.690, outperforming both NetMHCIpan-4.3EL (PR-AUC=0.543, TOP20%-PPV=0.592) and NetMHCIpan-4.3BA (PR-AUC=0.246, TOP20%-PPV=0.105). These results underscore the superiority of our model in accurately predicting peptide-MHC binding affinities across both MHC-I and MHC-II molecules, as detailed in **Figures 2A–D** (**Supplementary File 1**).

Additionally, we conducted comparative experiments for the MHC-I task using CcBHLA (24) and xTrimoPGLM (25). xTrimoPGLM is a large language model with 100 billion parameters, and its downstream tasks include peptide-HLA/MHC affinity prediction. In this comparison experiment, we used the same training set (575K), validation set (144K), and test set (171K) as CcBHLA and xTrimoPGLM. The test results were measured using Receiver Operating Characteristic - Area Under the Curve (ROC-AUC), OmniMHC yields an ROC-AUC of 98.35%, higher than 95.00% of CcBHLA scoring, and 96.68 of xTrimoPGLM, indicating OmniMHC outperforms the baseline methods.

### Application of OnmiMHC in TCGA tumor samples

We employed the dataset obtained by Xia et al. in their study, specifically utilizing **Supplementary Table S7** provided in their **Supplementary Materials** (26). Xia et al.’s data were obtained through the integration of clinical samples, bioinformatics tools, and experimental validation. Specifically, they initially gathered genomic information of tumor samples and patients’ HLA allele genotypes from various sources, including clinical collaboration projects and public databases such as The Cancer Genome Atlas (TCGA). Subsequently, they used a series of bioinformatics algorithms to predict potential novel antigenic peptide segments and experimentally validated the MHC binding capabilities of these peptides through IC50 binding affinity assays and cell stability experiments.

**Figure 3:**
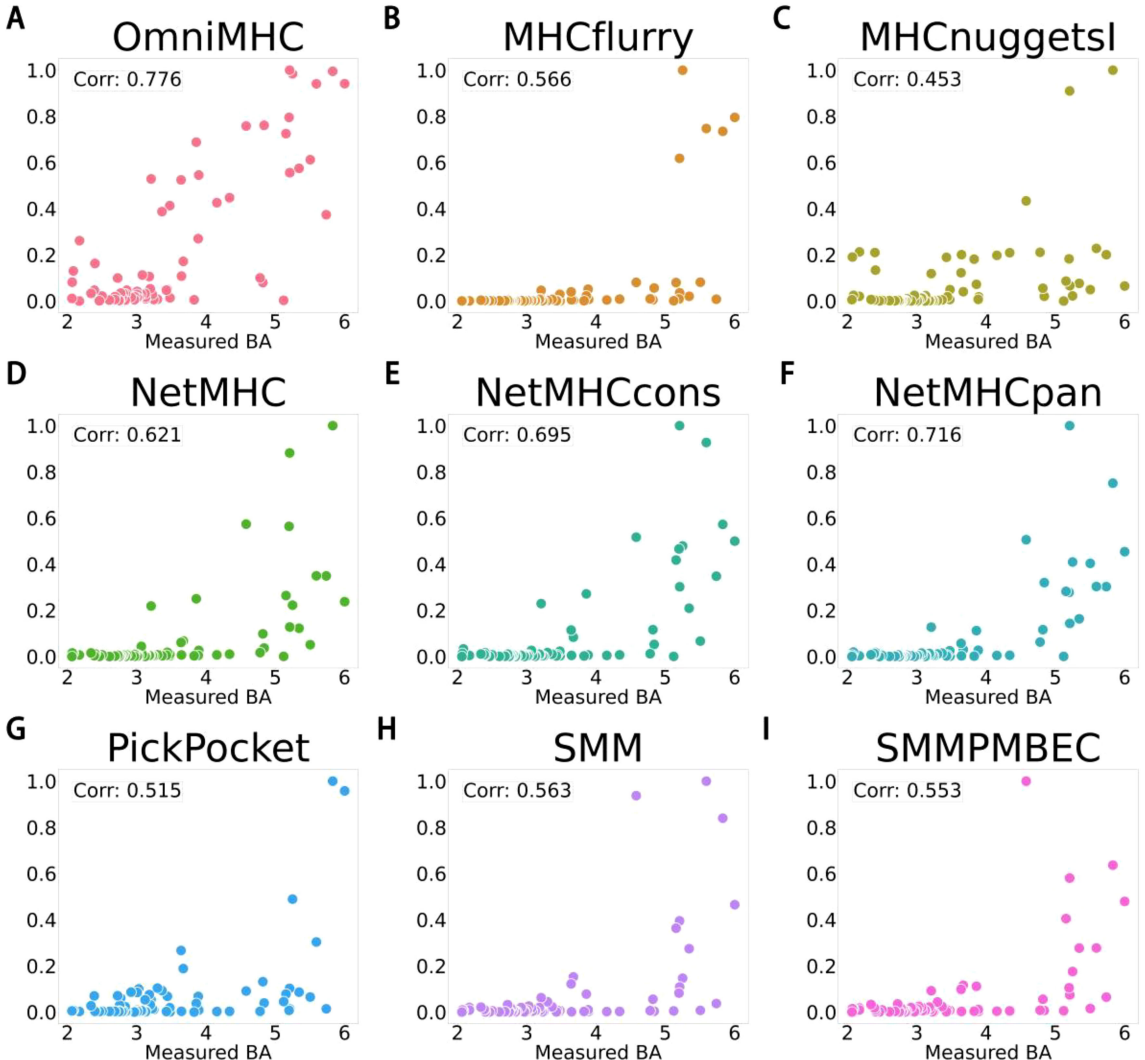
Description of scatter plot for the relationship between BA values of various models and their corresponding predicted values. **(A)** represents OnmiMHC, **(B)** represents MHCflurry (15), **(C)** represents MHCnuggets (16), **(D)** represents NetMHC (27), **(E)** represents NetMHCcons (13), **(F)** represents NetMHCpan (14), **(G)** represents PickPocket (23), **(H)** represents SMM (28), and **(I)** represents SMMPMBEC (29). x-axes is “Measured BA”, y-axes is “Predicted BA”.

We tested the OnmiMHC model on this dataset and conducted comparative experiments with other models. Here, we validated the performance using the Pearson correlation coefficient, and the results demonstrated that OnmiMHC exhibited the highest correlation coefficient of 0.78.as detailed in **Figure 3**(**Supplementary File 3**). This was tested on an independent test set, which is separated from the training dataset. As shown in **Figure 3**, different distributions of predicted values across different BA values can be noted. Particularly notable is the concentration of scatter points for the OnmiMHC model compared to all other models, indicating its superior predictive capability. This result further confirms the significant application potential of the OnmiMHC model in the development of tumor vaccines. By accurately predicting the binding of MHC molecules with tumor-specific antigens, OnmiMHC can facilitate the design and optimization of personalized tumor vaccines, enhancing the specificity and effectiveness of treatment. The model enables rapid screening of potential immunogens, accelerating the discovery and development of tumor immunogens.

### Application of OnmiMHC in EpiScan data

The EpiScan (30) technology represents a significant breakthrough in MHC class I ligand identification through an innovative screening process and advanced cell engineering. This technique utilizes an initial pool of over 100,000 synthetic peptides and specific cell lines modified with CRISPR-Cas9 technology (31, 32) to eliminate the interference of endogenous peptides, allowing for the exclusive expression of exogenous peptides. These exogenous peptides are transfected into cells via lentiviral vectors and subjected to high-throughput screening using flow cytometry. Subsequently, peptide identification is carried out through genomic DNA extraction, PCR amplification, and next-generation sequencing (33), resulting in a substantial dataset of peptide-allele binding interactions.

**Figure 4:**
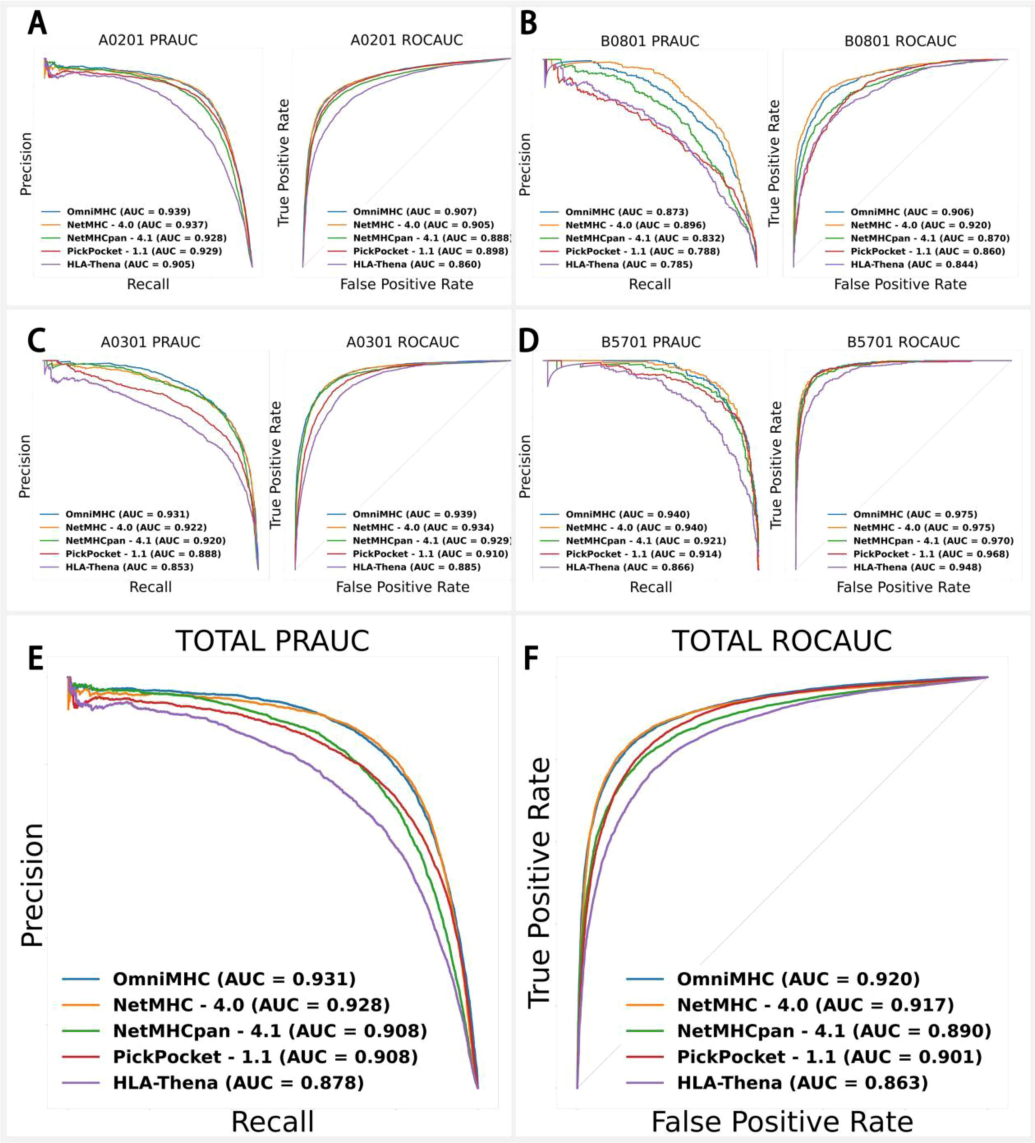
Application of OnmiMHC to EpiScan data with comparison of OnmiMHC with other models for each allele. **(A)** for A0201, **(B)** for A0301, **(C)** for B0801, and **(D)** for B5701. Each subfigure includes a comparison of OnmiMHC with other models using PR-AUC and ROC-AUC metrics. **(E)** shows PR-AUC results for various models, and **(F)** shows ROC-AUC results for the same models. The overall efficacy of OnmiMHC in predicting peptide-allele binding was evaluated by comparing results from multiple models across all alleles.

The EpiScan dataset consists of four alleles: B0801 (3,262 samples), B5701 (2,121 samples), A0301 (7,277 samples), and A0201 (19,205 samples). Ensuring no overlap between the test and training sets, we conducted comparative experiments using NetMHC-4.0, NetMHCpan-4.1, PickPocket-1.1, and HLA-Thena (19) against OnmiMHC. Given the relatively balanced ratio of positive to negative samples in this dataset, we employed PR-AUC and ROC-AUC metrics for more precise model performance evaluation. The results indicated that OnmiMHC performed exceptionally well for the A0201 (**Figure 4A**), B0801 (**Figure 4B**), A0301 (**Figure 4C**), B5701 (**Figure 4D**) alleles: for B5701, PR-AUC=0.940 and ROC-AUC=0.975; for A0301, PR-AUC=0.931 and ROC-AUC=0.939; and for A0201, PR-AUC=0.939 and ROC-AUC=0.907 (**Figure 4A**). Overall, across these four allele datasets, OnmiMHC achieved PR-AUC=0.931 and ROC-AUC=0.920, outperforming NetMHC-4.0, NetMHCpan-4.1, PickPocket-1.1, and HLA-Thena, establishing itself as the optimal model (**Figures 4E**, **F**) (**Supplementary File 4**).

### Application of OnmiMHC in uterine corpus endometrial carcinoma cancer tumor

We are conducting Cohort Frequency Peptide Analysis using the TCGA Uterine Corpus Endometrial Carcinoma(UCEC) dataset (34). Specifically, we utilize peripheral blood DNA-seq, tumor tissue DNA-seq, and RNA-seq data. Initially, we align and detect mutations in sequences using Burrows-Wheeler Aligner (BWA) (35) in conjunction with Samtools (36, 37). Subsequently, we annotate mutations using the Genome Analysis Toolkit (GATK) (38), including tools such as Variant Effect Predictor (VEP) (39) or ANNOVAR (40). The analysis identified the following mutation types: Silent, Missense Mutation, Splice Region, Frame Shift Del, Nonsense Mutation, In Frame Del, 3’ Flank, RNA, Frame Shift Ins, Intron, Splice Site, 5’ Flank, Nonstop Mutation, and Translation Start Site. From these, we selected Missense Mutation, Nonsense Mutation, Frame Shift Del, Frame Shift Ins, Splice Site, Intron, and Nonstop Mutation for further analysis.

**Figure 5:**
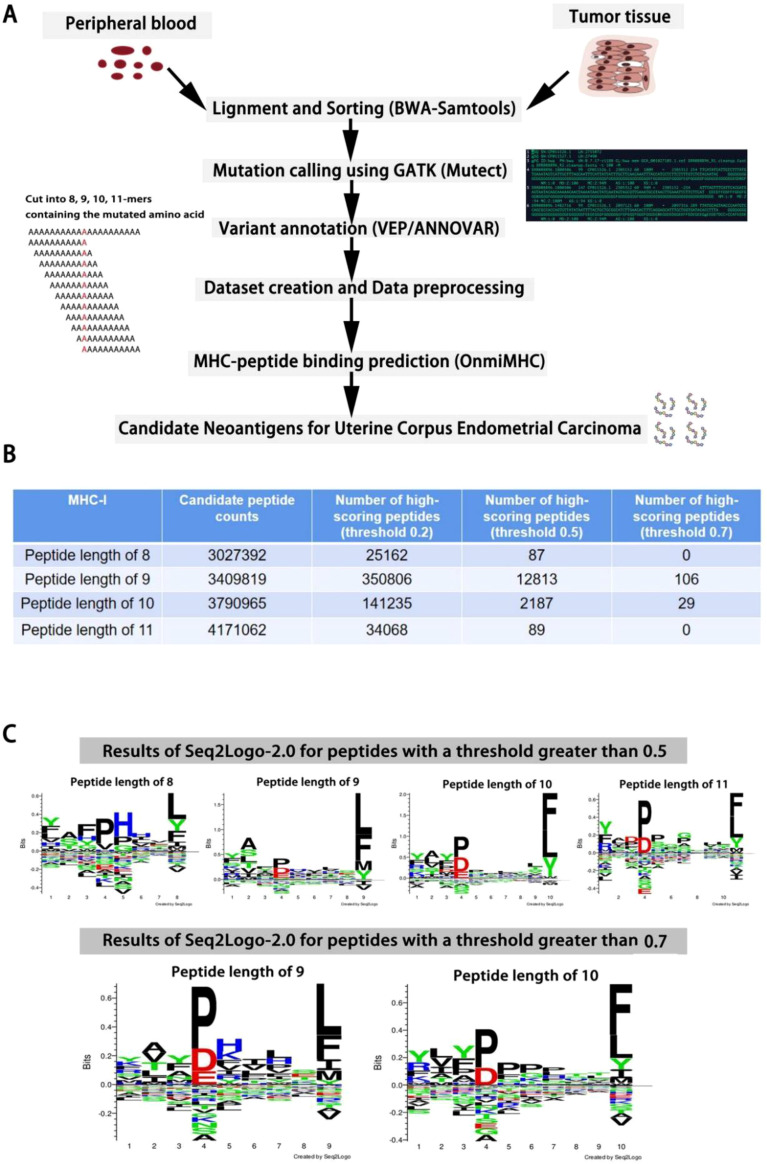
OnmiMHC application in UCEC. **(A)** Process flowchart for generating data, preprocessing, and predicting candidate neoantigens using OnmiMHC. **(B)** The number of candidate peptides and high-scoring peptides predicted by OnmiMHC. **(C)** Seq2logo analysis of high-scoring peptides identified above thresholds 0.5 and 0.7.

The data preprocessing steps included: 1. Removing peptides with missing values. 2. Eliminating records where the post-mutation peptide sequence remained unchanged. 3. Deduplicating the data. These steps ensured the accuracy and reliability of the analysis (**Figure 5A**). Next, we used sliding windows of lengths 8, 9, 10, and 11 to extract candidate peptides containing mutation sites. This resulted in datasets of 3,027,392 peptides of length 8, 3,409,819 peptides of length 9, 3,790,965 peptides of length 10, and 4,171,062 peptides of length 11 (**Figure 5B**). We combined these peptides with 20 common human alleles and used OnmiMHC for prediction. Finally, we identified high-scoring peptides by setting appropriate thresholds. These peptides can be designed into highly translatable mRNA sequences, which are then delivered into the human body via mRNA delivery systems. Since the candidate peptides are specific, this could be used to develop a targeted cancer vaccine (**Figure 5C**).

Finally, we used the average scores from 20 alleles to select the high-scoring peptides, applying three thresholds of 0.2, 0.5, and 0.7. The choice of these thresholds is based on a trade-off between precision and recall. A threshold of 0.7 provides a higher precision but leads to a decrease in recall, whereas a threshold of 0.2 results in a higher recall at the cost of precision. The threshold of 0.5 strikes a balance between the two, offering a reasonable tradeoff. Ultimately, we performed motif analysis using seq2logo (41) on peptides with average scores above the 0.5 and 0.7 thresholds. The results showed that the fourth residue P and the terminal residues L and F had higher bits in the high-scoring peptides.

### Model ablation studies on OnmiMHC

To evaluate the contribution of different components in the OnmiMHC model, we performed ablation studies on the different modules in OnmiMHC. Specifically, we assessed the impact of removing the CBAM attention mechanism, the 1DCNN_BiLSTM module, and the BLOSUM encoding individually. The performance of the ablated models was compared using PR-AUC and ROC-AUC metrics. We used the downstream task dataset of the MHC-I task from xTrimoPGLM. The model ablation experiments were conducted on the same training, testing, and validation sets.

- No CBAM: This variant excludes the CBAM attention mechanism while retaining the 1DCNN_BiLSTM module and 2DCNN and blosum encoding.
- No 1DCNN_BiLSTM: This variant excludes the 1DCNN_BiLSTM module while retaining the CBAM attention mechanism and 2DCNN and blosum encoding.
- No BLOSUM Encoding: This variant excludes the blosum encoding while retaining the CBAM attention mechanism and 2DCNN and the 1DCNN_BiLSTM module.

**Figure 6:**
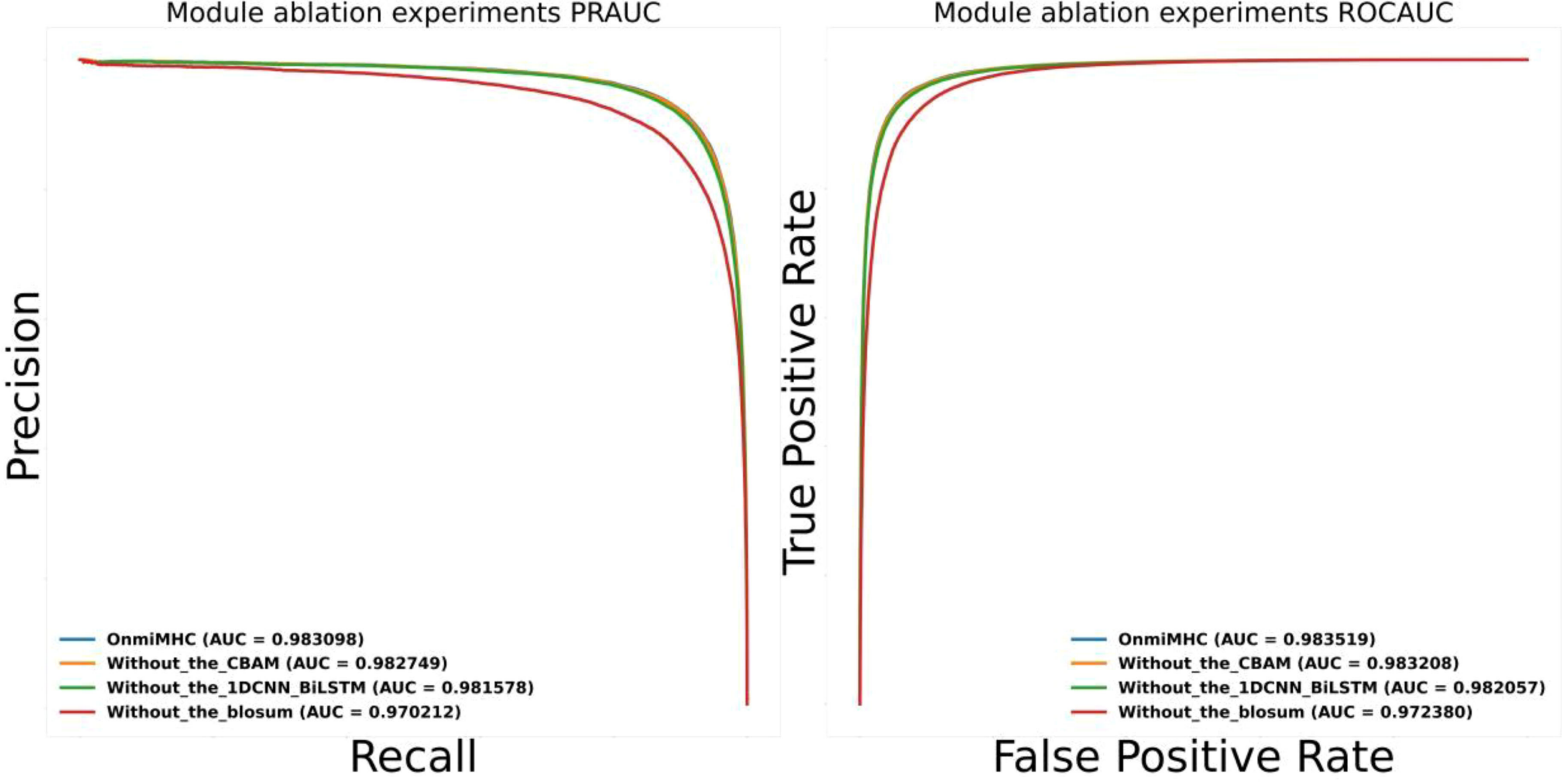
OnmiMHC ablation study results. This figure presents the performance comparison of the OnmiMHC model and its ablated variants in terms of AUC metrics. The blue line represents the baseline OnmiMHC model, incorporating all components. The orange line shows the performance without the CBAM attention mechanism. The green line indicates the performance without the 1DCNN_BiLSTM module. The red line represents the performance without BLOSUM encoding.

As shown in **Figure 6**, both the PR-AUC and ROC-AUC values are plotted. The results demonstrate that each component significantly contributes to the model’s predictive accuracy, as the removal of any single module leads to a noticeable decline in performance. To further investigate the contribution of each component, the CBAM mechanism plays a crucial role in capturing long-range dependencies and attention distribution across sequences, which is especially beneficial for identifying key features in complex sequence patterns. The 1DCNN_BiLSTM module is essential for feature extraction and learning temporal patterns, effectively capturing both local patterns in amino acid sequences and long-term dependencies. The BLOSUM encoding provides richer biological information about amino acid substitutions, which is crucial for the model’s performance when handling protein sequences.

## Discussion

In this study, we introduced the OnmiMHC model, a novel machine learning framework for predicting antigen peptide presentation by MHC Class I and II molecules. Our model integrates large-scale mass spectrometry data with other relevant data types, showcasing superior performance in both MHC-I and MHC-II prediction tasks. This discussion will focus on the implications, strengths, limitations, and future directions of our research.

The ability to accurately predict peptide-MHC binding is crucial for understanding immune responses and identifying potential immunogenic peptides. The superior performance of OnmiMHC, demonstrated through high PR-AUC and ROC-AUC scores, offers significant advancements in this area. By outperforming established models such as NetMHCpan-4.1 and NetMHCIIpan-4.3, OnmiMHC provides a more reliable tool for predicting peptide presentation, which is essential for the development of personalized immunotherapies and tumor vaccines. The improved accuracy and predictive capabilities of OnmiMHC can expedite the screening and evaluation of potential vaccine candidates, thereby reducing the time and resources required for tumor vaccine development.

One of the key strengths of OnmiMHC is its innovative design that integrates multimodal feature fusion and combines two-dimensional convolutional kernels with one-dimensional ones. This approach, along with the use of a BiLSTM for sequence information extraction and CBAM for feature attention, allows the model to comprehensively consider features from multiple perspectives. Additionally, the iterative data preprocessing method enhances the quality and robustness of the training data, leading to improved model performance. Our comparative experiments highlight OnmiMHC’s ability to generalize well across different datasets and alleles, reinforcing its potential utility in practical applications.

Despite the advancements made by OnmiMHC, several limitations need to be acknowledged. First, while our model showed a high predictive accuracy for MHC-II tasks, which is better than existing models, is still lower compared to MHC-I tasks. This indicates a need for further refinement in handling the complexities associated with MHC-II molecules. The difference in task complexity is one contributing factor: the peptide binding length for MHC-I typically ranges from 8 to 10 amino acids, whereas for MHC-II, it usually ranges from 13 to 17 amino acids. This results in a significantly larger feature space for MHC-II tasks, thereby increasing the complexity of the task. Furthermore, the differences in training data size also play a role. Currently, the publicly available data for MHC-I is far more abundant than for MHC-II. As such, there remains substantial room for improvement in MHC-II tasks using the OnmiMHC, especially as more MHC-II sample data becomes available. We expect the model’s performance to further improve with the availability of larger datasets. Second, the iterative data preprocessing approach, although effective, may inadvertently exclude relevant peptide sequences, potentially affecting the model’s comprehensiveness.

Future research should aim to enhance the OnmiMHC model by integrating additional data types such as structural information and peptide-MHC binding kinetics. The interaction between MHC molecules and peptides depends not only on the peptide sequence but also on the structural interplay between the peptide and MHC. Introducing three-dimensional structural data for both MHC molecules and peptides, derived from protein structure prediction tools like AlphaFold or experimental data from protein databases like PDB, could improve the model. By combining this structural information with existing sequence data, the model could more accurately predict MHC-peptide binding potential. Furthermore, many current models focus on the static state of peptide-MHC binding, neglecting the dynamic nature of the interactions. Incorporating peptide-MHC binding kinetics data, such as association and dissociation rate constants, could allow the model to better reflect the temporal aspects of peptide-MHC interactions, which are crucial in certain clinical applications where binding kinetics may be more significant than static binding strength.

To improve the model’s ability to handle a broader range of peptide sequences, especially those with mutations or modifications, future research could introduce more diverse peptide sequences. Advanced sequence representation methods, such as self-attention mechanisms from Transformer architectures, could be applied to capture more complex binding patterns and enhance the model’s generalization capacity. Additionally, refining the representation of negative samples, particularly in MHC-II tasks, is essential. Current methods often generate negative samples randomly, which may not represent the true distribution of negative samples in biological systems. More diverse methods for generating negative samples, such as using peptide-MHC interaction models or analyzing unbound peptides in experimental datasets, could improve the model’s accuracy in handling negative samples. Finally, the application of transfer learning techniques could be a promising direction. By pretraining the model on a large peptide-MHC binding dataset and then fine-tuning it on related tasks, such as MHC-II tasks, the model could adapt more effectively to new datasets and experimental conditions. Additionally, cross-domain transfer learning could enable the model to transfer knowledge learned from MHC-I tasks to MHC-II tasks or from peptide prediction models to other protein-molecule interaction tasks, accelerating the model training and enhancing its generalization ability across various biomedical domains.

Another promising direction is the application of OnmiMHC in real-life scenarios, such as clinical trials of personalized immunotherapy. In the aforementioned description of the Application of OnmiMHC in UCEC, we utilized OnmiMHC to identify high-binding probability candidate peptide segments associated with UCEC tumors. These peptides can serve as new antigen data to collaborate with experimental biologists and clinical physicians to validate the model’s predictions in practical settings and optimize its performance based on real-world feedback. In readl-world application, the tumor microenvironment plays a crucial role in the tumor development and immune responses, and it is particularly relevant in the context of tumor vaccine development (42, 43).

The OnmiMHC model represents a significant step forward in the prediction of peptide-MHC binding, offering improved accuracy and predictive capabilities over existing models. By addressing the current challenges in MHC-peptide binding prediction, OnmiMHC provides valuable insights and tools for understanding immune responses, identifying immunogenic peptides, and advancing personalized immunotherapy. Continued refinement and application of this model hold great promise for enhancing the efficiency and effectiveness of tumor vaccine development and other immunotherapeutic strategies.

## Materials and methods

### OnmiMHC model architecture

OnmiMHC employs two encoding methods: BLOSUM62 (17) and one-hot encoding. BLOSUM62 is a protein sequence alignment algorithm widely used in bioinformatics and computational biology (44–46). It converts amino acids in protein sequences into representative numbers based on their chemical properties and evolutionary similarities, allowing neural networks to better capture complex biological patterns. By considering amino acid substitution scores, BLOSUM62 provides a more biologically meaningful encoding, which is particularly advantageous when dealing with protein sequence alignment.

In contrast, one-hot encoding represents each amino acid with an N-dimensional vector, where N is the total number of amino acids. In this vector, only one element is set to 1, and the remaining elements are set to 0, with the position of the 1 corresponding to the current amino acid. This encoding method is simple and intuitive, easy to implement, and ensures that each amino acid is treated as a distinct entity. While one-hot encoding does not capture evolutionary or chemical similarities, it allows the model to easily learn the structural features of each individual amino acid, making it a useful tool for straightforward sequence analysis tasks.

Comparing the two methods, BLOSUM62 is generally more informative as it accounts for amino acid substitutions, offering a richer representation that may be crucial for capturing deeper biological relationships. On the other hand, one-hot encoding is computationally less demanding and may be preferable when simplicity or computational efficiency is a priority. By using both encoding strategies in OnmiMHC, we combine the advantages of both approaches, allowing the model to leverage both detailed biological context and simple, interpretable representations of amino acids.

Once the sequence encoding is complete, OnmiMHC uses two different types of neural network models, 1D-CNN-LSTM and 2D-CNN, to decode and extract temporal and spatial local features. After convolution, the CBAM attention mechanism is applied to re-attend to the features (47, 48). 1D-CNN-LSTM is a hybrid model that combines a 1D convolutional neural network and a long short-term memory network (49). It can capture both the temporal information and local features of sequences. By applying 1D convolution to the sequence, the model detects local patterns, while the LSTM captures long-term dependencies within the sequence. In this context, 1D-CNN-LSTM transforms the encoded sequence into temporal features, providing a better understanding of the sequence’s context.

1D convolution is represented as:


(1) $${\text{y}}_{i}=\text{σ}\left(\sum_{j=0}^{m-1}W_{j}X_{i+j}+b\right)$$

where ${\text{y}}_{\text{i}}$ is the i-th element of the output, σ is the activation function, ${\text{W}}_{\text{j}}$ is the weight of the j-th convolution kernel, ${\text{X}}_{\text{i}+\text{j}}$ is the i+j-th element of the input sequence, and b is the bias.

LSTM is represented as:


$$f_{t}=\sigma \left(W_{f}\cdot \left[h_{t-1},x_{t}\right]+b_{f}\right)$$

$$i_{t}=\sigma \left(W_{i}\cdot \left[h_{t-1},x_{t}\right]+b_{i}\right)$$

(2) $$\;\;\;\;\;\;\;\;\;\;\;\;\;o_{t}=\sigma \left(W_{o}\cdot \left[h_{t-1},x_{t}\right]+b_{o}\right)$$

$${\tilde{C}}_{t}=\text{tanh}\left(W_{c}\cdot \left[h_{t-1},x_{t}\right]+b_{c}\right)$$

$$C_{t}=f_{t}\cdot C_{t-1}+i_{t}\cdot {\tilde{C}}_{t}$$

$$h_{t}=o_{t}\cdot \text{tanh}\left(C_{t}\right)$$

where ${\text{f}}_{\text{t}}$ is the forget gate, ${\text{i}}_{\text{t}}$ is the input gate, ${\text{o}}_{\text{t}}$ is the output gate, ${\tilde{\text{C}}}_{\text{t}}$ is the candidate unit state, ${\text{C}}_{\text{t}}$ is the unit state, and ${\text{h}}_{\text{t}}$ is the output.

2D-CNN is a commonly used convolutional neural network designed to process image data with a planar structure (50, 51). OnmiMHC rearranges sequences into a 2D image matrix format to utilize 2D-CNN for extracting planar local features from the sequences. 2D-CNN can detect planar patterns within the sequences, thereby extracting their planar local features.

The 2D convolutional neural network is represented as:


(3) $$y_{i,j}=\sigma \left(\sum_{k=1}^{K}\sum_{l=1}^{L}w_{k,l}\cdot x_{i+k,j+l}+b\right)$$

where ${\text{y}}_{\text{i},\text{j}}$ is the i,jth element of the output, σ *is the activation function*, ${\text{w}}_{\text{kl}}$ is the weight of the k,lth convolution kernel, ${\text{x}}_{\text{i}+\text{k},\text{j}+\text{l}}$ is the i+k,j+lthe lement of the input matrix, K and L are the sizes of the convolution kernel, and b is the bias.

By using 2D-CNN, OnmiMHC can obtain different local feature representations compared to 1D-CNN-LSTM. By combining these two types of neural network models, OnmiMHC can capture both temporal and planar local features of the sequences, resulting in a more comprehensive sequence feature representation.

CBAM (Convolutional Block Attention Module) (52) is an attention mechanism for convolutional neural networks. It enhances the network’s feature representation capability by introducing channel attention and spatial attention, thereby improving performance in tasks like image recognition and object detection. CBAM can be inserted into existing convolutional neural networks, and it improves the model’s performance by learning important feature positions and feature channels. CBAM consists of two main modules: the Channel Attention Module and the Spatial Attention Module.

The Channel Attention Module obtains attention weights for the channel dimension by applying global average pooling and global max pooling to the input feature map. These weights are then applied to the input feature map to highlight important feature channels.

The channel attention weights are calculated by global average pooling and global maximum pooling:


(4) $$\;\;\;\;\;\;\;\;\;\;M_{c}\left(F\right)=\sigma \left(MLP\left(AvgPool\left(F\right)\right)+MLP\left(MaxPool\left(F\right)\right)\right)$$

where ${\text{M}}_{\text{c}}\left(\text{F}\right)$ is the channel attention weight σ is the sigmoid activation function, MLP is a multi-layer perceptron,AvgPool and MaxPool are global average pooling and global maximum pooling respectively.

The Spatial Attention Module obtains attention weights for the spatial dimension by applying average pooling and max pooling along the channel dimension of the input feature map. These weights are then applied to the input feature map to highlight important spatial locations.

The spatial attention weights are calculated by average pooling and max pooling in the channel dimension:


(5) $$\;\;\;\;\;\;\;\;\;\;M_{s}\left(F\right)=\sigma \left(Conv\left(\left[AvgPool\left(F\right);MaxPool\left(F\right)\right]\right)\right)$$

where ${\text{M}}_{\text{s}}\left(\text{F}\right)$ is the spatial attention weight, σ is the sigmoid activation function, Conv is the convolution operation,[;]; represents the concatenation of feature maps,AvgPool and MaxPool are global average pooling and global maximum pooling respectively.

The feature map after combining these two modules is expressed as:


(6) $$F^{\prime }=M_{s}\left(F\right)*F$$

(7) $$F^{\prime \prime }=M_{s}\left(F^{\prime }\right)*F^{\prime }$$

where ${\text{F}}^{\prime }$ is the feature map after applying channel attention, ${\text{F}}^{\prime \prime }$ is the feature map after applying spatial attention.

By combining the Channel Attention Module and the Spatial Attention Module, CBAM effectively enhances the network’s feature representation capability, thereby improving the model’s performance in various computer vision tasks (**Supplementary Method 1**).

In this study, OnmiMHC first concatenates peptide sequences with MHC molecule sequences and performs one-hot encoding. Then, OnmiMHC inputs these encoded features into both 1D-CNN-LSTM and 2D-CNN models for decoding. Additionally, OnmiMHC separately performs one-hot encoding and BLOSUM62 encoding for the peptide sequences. Finally, OnmiMHC merges all encoding and decoding features, and reduces dimensionality through fully connected layers to predict the binding affinity or probability between peptides and MHC. Specifically, binding affinity prediction is utilized for pre-training strategy. First, the OmniMHC pre-trained model is trained using the BA dataset. Next, the pre-trained model is used to clean the data in the EL dataset. Finally, the optimized datasets are combined to train the final OmniMHC model.

As for the training parameters, the batch size of the MHC-I model is 40,000, while MHC-II model’s batch size is 20,000. The learning rate for both models is set to 0.0001, and they use a CosineAnnealingLR scheduler with T_max=30. The optimizer is AdamW, and we train the models for 30 epochs.

### Model pre-training using the BA dataset.

In this step, we curated the BA dataset. For MHC-I tasks, we collected five-fold BA datasets from NetMHCpan-4.1. For MHC-II tasks, we gathered five-fold BA datasets from NetMHCIIpan-4.0. We utilized the OnmiMHC model architecture for regression training on these datasets using the Mean Squared Error (MSE) loss function, where the training labels are score values. The specific representation is as follows: first, take the natural logarithm of the IC50 value, then divide this logarithmic value by the natural logarithm of 50000, and finally subtract this ratio from 1.

The specific formula is:


(8) $$\;\;\;\;\;\;\;\;\;\;\;\;\;\;\;\;\;\text{score}=1-\left(\frac{\text{ln}\left(IC50\right)}{\text{ln}\left(50000\right)}\right)$$

### Data preprocessing and label generation

In the second step, we utilize the OnmiMHC pre-training model obtained from the first step to preprocess Mass Spectrometry Eluted Ligand Single Allele (MS ELs-SA) and Mass Spectrometry Eluted Ligand Multi Alleles (MS ELs-MA) datasets. This preprocessing aims to enhance data quality and representation capability. Specifically, the OnmiMHC pre-training model predicts peptide-MHC binding and outputs binding affinity scores.

For the MS ELs-MA dataset, the OnmiMHC pre-training model predicts each combination sample and assigns the allele with the highest score as its label, thereby converting the multi-allele binding data into a single-allele dataset, MS ELs-SA. It’s noteworthy that both MS ELs-SA and MS ELs-MA datasets originate from experiments involving peptide elution from MHC molecules. Such experiments involve eluting antigenic peptides from MHC molecules using acidic solutions or other methods, followed by identification and analysis of the peptides via mass spectrometry or other techniques to obtain peptide sequences. Consequently, these experimental methods only yield positive samples capable of binding. Negative samples are typically randomly selected peptide sequences from the human body, although this practice lacks rigor. To address this issue, the OnmiMHC pre-training model scores these negative samples and removes those with higher binding scores, ensuring more accurate representation of non-binding scenarios.

Finally, OnmiMHC merges the preprocessed MS ELs-SA, MS ELs-MA, and BA datasets to form a high-quality, large-scale dataset by maintaining the original data splits. This dataset not only contains information related to peptide-MHC binding events but also encompasses information relevant to previous steps in the biological antigen presentation pathway.

### Integration of all datasets to train the binding probability prediction model

In the third step, we preprocess the BA dataset by changing labels: IC50 values less than 500nm are set to 1 (positive samples), while those greater than or equal to 500nm are set to 0 (negative samples). With the curated datasets from previous steps, we now possess MS ELs-SA, MS ELs-MA, and BA datasets with labels of 0 and 1. We again utilize the OnmiMHC model architecture to train on these datasets.

Unlike the first step, we are now dealing with a classification task. We employ cross-entropy loss function and backpropagation algorithm to update neural network parameters (53). Cross-entropy loss function is a commonly used classification loss function, effectively assessing model prediction performance (54). Backpropagation algorithm updates neural network parameters by computing gradients, thus improving model prediction performance.

We employ 5-fold cross-validation to train and evaluate model performance, selecting the model with the minimum test set loss as the optimal model. Finally, we average the output results of the five Cross-validation models to obtain the final model output. This approach not only effectively utilizes all datasets but also mitigates model overfitting issues.

In summary, the process is akin to semi-supervised learning. We begin with the BA dataset, which contains continuous numeric labels. After training a regression model on this dataset, we use the model to score the negative samples from the EL dataset. Employing a greedy strategy, we selectively retain only the negative samples with the lowest scores. Finally, we integrate all datasets into classification labels using a threshold and train a classification model.

### Data curation

In this study, we utilized datasets generated by Birkir Reynisson and their colleagues These datasets combine public domain data on MHC binding affinity (BA) and mass spectrometry (MS) eluted ligands (EL). We obtained these datasets from the public web servers http://www.cbs.dtu.dk/services/NetMHCpan-4.1/ and http://www.cbs.dtu.dk/services/NetMHCIIpan-4.0/, covering a wide range of MHC class I and class II molecules (14).

For the MHC-I part, we also used datasets generated and published by the research team of Siranush Sarkizova and their colleagues. These datasets, obtained through high-resolution mass spectrometry (LC-MS/MS), include over 185,000 peptides eluted from cell lines expressing 95 different HLA-A, -B, -C, and -G alleles. They not only provide identification information of HLA binding peptides, but also detailed characteristics of peptides binding to HLA molecules, such as peptide length preferences, binding submotifs, and specific binding patterns for different HLA alleles (19).

For the MHC-II part, we used datasets generated by C. Garrett Rappazzoand their colleagues. These datasets were produced using an innovative yeast display platform that allows for the identification of an order of magnitude more unique MHC-II binding peptides compared to existing methods (1).

Detailed specifics of the datasets, including the MHC-II alleles used, peptide lengths, and related binding affinity data, can be found in the original publications. Our data compilation aims to maximize the utilization of these publicly available datasets to advance the field of MHC antigen prediction.

## Data Availability

The data and source code of OnmiMHC are freely available at https://github.com/caihaihua057200/OnmiMHC for academic use.
