# Supplementary material for: OnmiMHC: a machine learning solution for UCEC tumor vaccine development through enhanced peptide-MHC binding prediction
Source: Front Immunol. 2025 Feb 28;16:1550252. doi: 10.3389/fimmu.2025.1550252 (PMC11906482; doi:10.3389/fimmu.2025.1550252)
Supplement: Supplementary file 5 [file Supplementaryfile5.docx]

**Supplementary information**

**Supplementary Method 1: OnmiMHC Model Structure**

The OnmiMHC model is designed to predict peptide-MHC binding affinity and probability. The model structure is illustrated in Supplementary Figure 1.

**Feature Encoding:**

Peptide sequences are concatenated with MHC molecule sequences.These concatenated sequences are encoded using one-hot encoding.Peptide sequences are also separately encoded using BLOSUM62.

**Model Architecture:**

The encoded features are processed through a combination of 1D-CNN-LSTM and 2D-CNN models.

The 1D-CNN-LSTM model captures sequential and spatial information from the concatenated sequences.

The 2D-CNN model, integrated with a CBAM (Convolutional Block Attention Module), further extracts and refines spatial features by focusing on important regions of the input. Both one-hot and BLOSUM62 encoded features are utilized to enhance the model's ability to capture diverse sequence representations.

**Feature Integration and Prediction:**

All features are merged into a unified representation. Dimensionality reduction is performed through fully connected layers.The final output layer predicts peptide-MHC binding affinity or probability. This comprehensive approach allows the OnmiMHC model to effectively integrate and process diverse sequence information, resulting in accurate predictions of peptide-MHC binding affinity and probability.


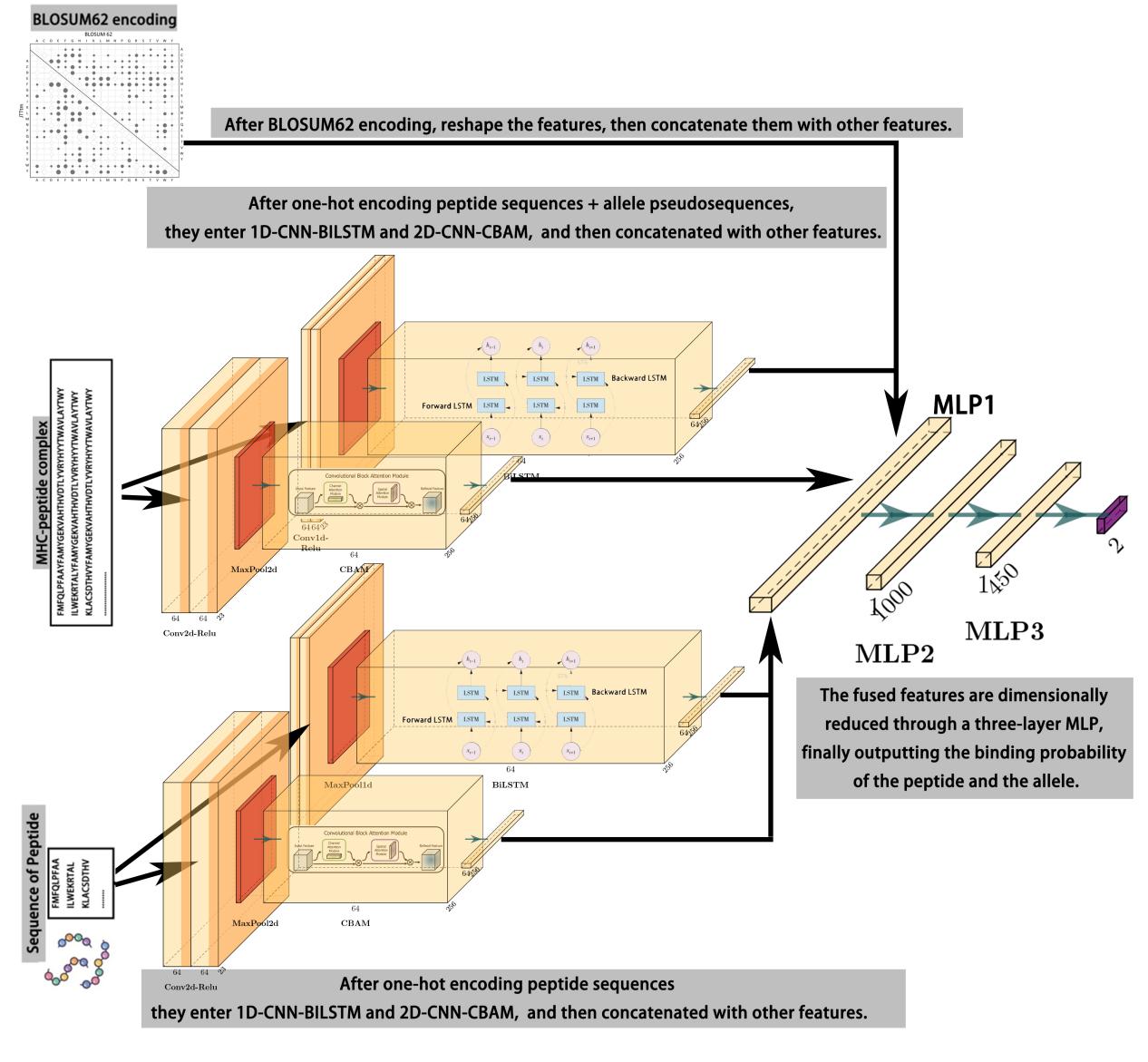


**Supplementary Figure 1 | OnmiMHC Model Structure**

OnmiMHC concatenates peptide sequences with MHC molecule sequences and encodes them using one-hot encoding. These features are processed by 1D-CNN-LSTM and 2D-CNN models. Additionally, one-hot and BLOSUM62 encodings are performed separately for peptide sequences. All features are merged and reduced in dimensionality through fully connected layers to predict peptide-MHC binding affinity or probability.

**Supplementary Method 2: Model Pan-Allel Test**

To ensure the model has strong generalization capabilities, we tested OnmiMHC using out-of-sample alleles. During the training phase for the MHC-I tasks, we manually excluded specific alleles such as A02:02 and C02:02 to ensure they were not included in the training set. The model was then tested on data containing these excluded alleles. For the MHC-II tasks, alleles such as DRB103:01 and DRB101:01 were excluded during training and used for testing the model.

**The test results demonstrated the generalization capabilities of OnmiMHC:**

- For the A*02:02 allele, the model achieved a PR-AUC of 0.993 and a ROC-AUC of 0.950.
- For the C*02:02 allele, the PR-AUC was 0.948 and the ROC-AUC was 0.994.
- For the DRB1*03:01 allele, the PR-AUC was 0.362 and the ROC-AUC was 0.816.
- For the DRB1*01:01 allele, the PR-AUC was 0.173 and the ROC-AUC was 0.692.

These results fully demonstrate the model's strong generalization capabilities (Supplementary_File_2).


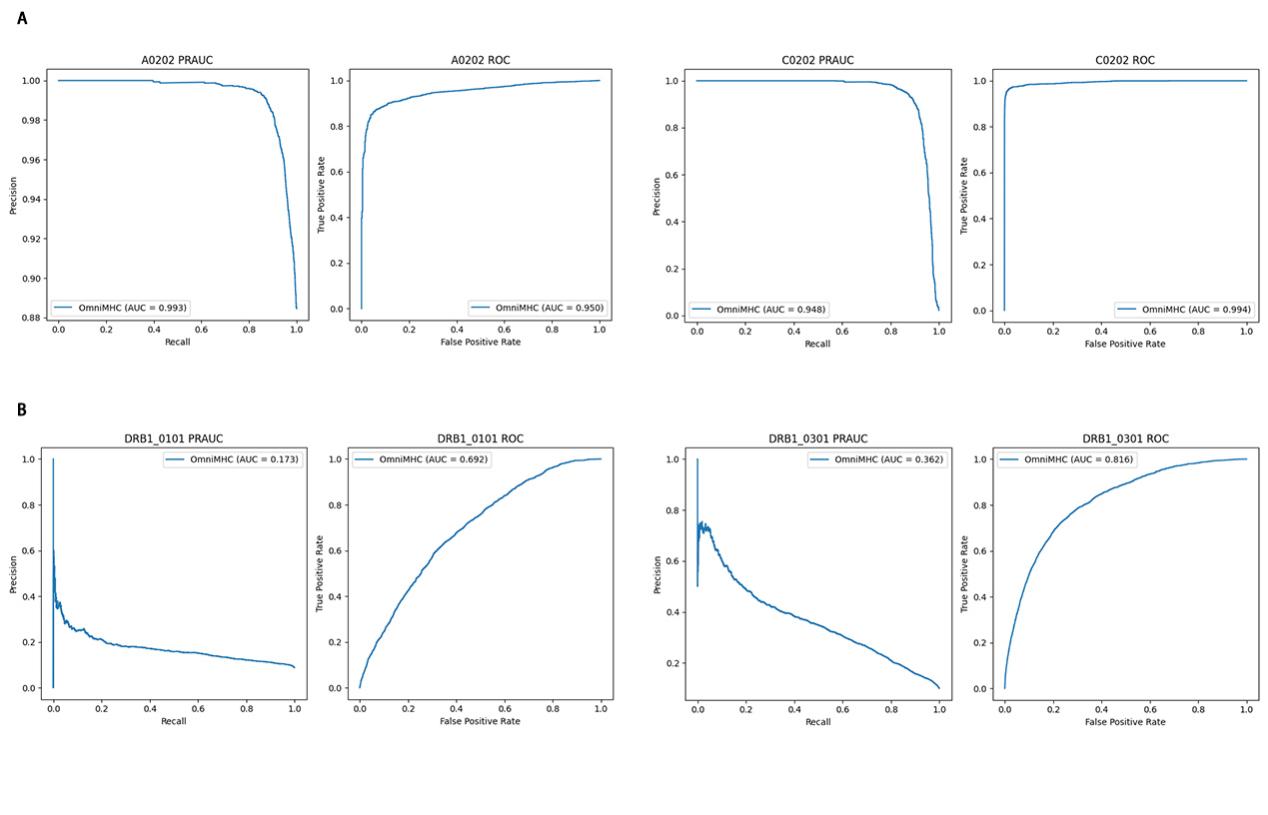


**Supplementary Figure 2 | OnmiMHC Pan-Allel Test**

The figure illustrates the results of the pan-allele test conducted to assess OnmiMHC's generalization capabilities across diverse alleles. Substantial PR-AUC and ROC-AUC scores were achieved for both MHC-I and MHC-II tasks, indicating the model's robustness in predicting peptide-MHC binding across various allele types.

[1] Chen, Bo, et al. "xTrimoPGLM: unified 100B-scale pre-trained transformer for deciphering the language of protein." *arXiv preprint arXiv:2401.06199* (2024).
